# The Impact of Basal Inflammatory Status on Post-CABG Atrial and Ventricular Ectopy and Remodeling Pathways

**DOI:** 10.3390/medicina61091545

**Published:** 2025-08-27

**Authors:** Dan-Alexandru Cozac, Cristina Somkereki, Adina Huțanu, Tunde Renata Nicoara, Alina Scridon

**Affiliations:** 1 Doctoral School of Medicine and Pharmacy, George Emil Palade University of Medicine, Pharmacy, Science, and Technology of Targu Mures, 540142 Targu Mures, Romania; dan-alexandru.cozac@umfst.ro; 2 Physiology Department, George Emil Palade University of Medicine, Pharmacy, Science, and Technology of Targu Mures, 540142 Targu Mures, Romania; 3 5th Medical Department, George Emil Palade University of Medicine, Pharmacy, Science, and Technology of Targu Mures, 540142 Targu Mures, Romania; cristina.somkereki@umfst.ro; 4 Emergency Institute for Cardiovascular Diseases and Transplantation of Targu Mures, 540136 Targu Mures, Romania; p.renata93@gmail.com; 5 Department of Laboratory Medicine, George Emil Palade University of Medicine, Pharmacy, Science, and Technology of Targu Mures, 540142 Targu Mures, Romania; adina.hutanu@umfst.ro; 6 Center for Advanced Medical and Pharmaceutical Research, George Emil Palade University of Medicine, Pharmacy, Science, and Technology of Targu Mures, 540142 Targu Mures, Romania

**Keywords:** biomarker, cardiac arrhythmias, coronary artery bypass, preoperative care

## Abstract

*Background and Objectives:* Premature atrial contractions (PACs) and premature ventricular contractions (PVCs) commonly occur after coronary artery bypass grafting (CABG) surgery, with frequent ectopics linked to atrial fibrillation risk and reduced heart function. While CABG-induced inflammation causes arrhythmogenic changes, the connection between preoperative inflammatory markers and postoperative ectopic burden has not been studied. Therefore, the aim of the present study is to evaluate the association between preoperative inflammatory biomarkers and postoperative atrial and ventricular ectopic burden, and to determine their influence on clinical outcomes following elective CABG procedures. *Materials and methods:* This study assessed preoperative plasma levels of highly sensitive C-reactive protein (hs-CRP), von Willebrand factor (vWF), transforming growth factor-β (TGF-β), interleukin (IL)-2, IL-1β, IL-6, IL-8, and vascular endothelial growth factor (VEGF) using the Multiplex technique in patients undergoing elective CABG. A continuous 24-h ECG Holter monitoring was performed one day before CABG, as well as on days 2, 3, and 4 post-CABG. The PACs and PVCs burdens were quantified, and correlations with clinical parameters were analyzed. *Results:* Preoperative plasma concentrations of vWF, TGF-β, and IL-8 exhibited significant positive correlations with postoperative PACs (*p* < 0.001, *p* = 0.03, and *p* < 0.001, respectively). Preprocedural hs-CRP, TGF-β, IL-6, and IL-8 levels showed significant positive associations with PVCs (*p* < 0.0001, *p* < 0.0001, *p* = 0.02, and *p* < 0.0001, respectively). However, none of the tested biomarkers could predict other postoperative outcomes, such as acute kidney injury, acute liver failure, duration of inotropic support, and days of hospitalization. *Conclusions:* Preoperative inflammatory biomarkers may serve as predictive tools for postoperative ectopic activity following CABG. Early identification of high-risk patients could enable prophylactic strategies and improve post-CABG outcomes.

## 1. Introduction

Cardiac arrhythmias represent one of the most common postoperative complications after coronary artery bypass grafting (CABG) surgery, significantly impacting the healthcare system and increasing the costs of postoperative medical care [1]. Despite major improvements in cardiac surgery strategies over the last decades, postoperative arrhythmias remain a major contributor to cardiovascular morbidity and mortality [2].

Premature atrial contractions (PACs) have traditionally been considered benign electrocardiographic findings following CABG [3]. However, a high burden of PACs has recently been associated with an increased risk of incident atrial fibrillation (AF) and other adverse outcomes, particularly stroke [4,5]. Premature ventricular complexes (PVCs) are also common postoperatively [6]. Although they do not seem to increase the risk of malignant ventricular arrhythmias and have a controversial prognostic value, frequent PVCs can affect patients’ short-term clinical outcomes by impairing ventricular performance [3]. Moreover, in patients with significantly reduced left ventricular ejection fraction (LVEF), a high PVC burden can result in higher rates of mortality [7].

The positive relationship between PACs and the subsequent risk of incident AF, as well as the association of frequent PVCs with impaired LVEF and hemodynamic complications post-CABG, emphasize the importance of early identification of patients at risk of developing atrial and ventricular ectopy after CABG [4,8]. Several patient-related clinical risk markers have been described in this regard. These include older age, reduced systolic function, left atrial enlargement, chronic heart failure, and obesity [9,10]. Surgery-related risk factors, including hemodynamic stress, ischemic injury due to cardiorespiratory bypass, cardioplegia, and cardiac surgery itself, are all associated with inflammation [3,11]. Myocardial ischemia during CABG significantly increases systemic inflammation and promotes the recruitment of inflammatory leukocytes [12]. Activation of the inflammatory cascade exerts detrimental effects on the heart tissue, promoting proarrhythmic electrical changes and ectopy [13]. Previous studies have suggested that several inflammatory markers may be useful for guiding post-surgery care, particularly for patients at risk of arrhythmic events [14]. Biomarkers such as highly sensitive C-reactive protein (hs-CRP), von Willebrand factor (vWF), transforming growth factor-*beta* (TGF-β), interleukin (IL)-6, and IL-7 have been associated with post-CABG arrhythmic complications [15,16]. Nonetheless, most of these studies have assessed the correlation between post-operative biomarker levels and arrhythmogenic events, especially AF and ventricular tachycardia.

To the best of our knowledge, no study to date has evaluated the relationship between preoperative biomarker levels and postoperative atrial and ventricular ectopic burden. Early identification of patients at high risk of post-CABG ectopic activity could enable stringent screening strategies and targeted prophylactic therapy, and could thus improve post-CABG outcomes.

Accordingly, the present study aimed to assess the impact of preoperative plasma levels of hs-CRP, vWF, TGF-β, IL-2, IL-1β, IL-6, IL-8, and vascular endothelial growth factor (VEGF) on postoperative atrial and ventricular ectopic activity in patients undergoing elective CABG procedures. The impact of postoperative ectopic arrhythmic burden on post-CABG outcomes was also assessed.

## 2. Materials and Methods

### 2.1. Study Population

Patients diagnosed with chronic coronary syndrome (CCS) according to the European Guidelines on Myocardial Revascularization and referred for elective CABG at the Emergency Institute for Cardiovascular Disease and Transplantation of Targu Mures were initially considered eligible [17]. The research protocol was in accordance with the Declaration of Helsinki and was approved by the local Ethics Committee of the University of Medicine and Pharmacy of Târgu Mureș, Romania (approval number 31/21.03/2016). To be included, patients had to be older than 18 years, in sinus rhythm, and hemodynamically stable at the time of evaluation. Subsequently, only those with a comparable degree of coronary artery disease were included to avoid potential confounding factors related to disease severity. Informed consent was obtained from all participants involved in the study.

To further minimize confounding, we excluded patients in whom complete revascularization was not achieved, patients with a prior history of AF or documented ventricular arrhythmias, those scheduled for concomitant valve replacement or other surgical interventions, and those receiving ongoing treatment with class I, III, or IV antiarrhythmic drugs. Patients with mitral stenosis of any degree, more than mild mitral regurgitation, left ventricular ejection fraction below 40%, as well as those with systemic and/or autoimmune inflammatory diseases, were also excluded. Finally, patients receiving chronic anti-inflammatory therapy, including nonsteroidal anti-inflammatory drugs, corticosteroids, or disease-specific immunomodulatory treatment, were not considered for the study. Patients were followed throughout their entire hospitalization for CABG, with investigations conducted and data collected preoperatively, intraoperatively, and postoperatively, until discharge.

### 2.2. Evaluated Characteristics [17]

Major cardiovascular risk factors were evaluated, including age, male gender, smoking status, history of diabetes mellitus, and previous myocardial infarction. The components of the CHA_2_DS_2_-VASc score (congestive heart failure, hypertension, age ≥ 75 years, diabetes mellitus, stroke, vascular disease, age 65–74 years, female sex) and SYNTAX II score were calculated for each patient. The history of statin, renin-angiotensin-aldosterone system (RAAS) inhibitors, and beta-blocker use was collected for each patient. Echocardiographic characteristics, including LVEF and left atrium diameter and area, were also noted. For each patient, continuous 24 h ECG Holter monitoring was performed one day before CABG, as well as on days 2, 3, and 4 post-CABG, and the PACs and PVCs burdens were quantified by reporting the percentage of ectopic beats per hour. All patients were managed postoperatively according to standard institutional protocols. Pre-existing beta-blockers or calcium channel blockers were continued, while no prophylactic class I, III, or IV antiarrhythmic medications were administered during the observation period. Peripheral venous blood samples were obtained one day before CABG, prior to any intervention or administration of any anti-inflammatory medication. Plasma was isolated from whole blood collected in EDTA-anticoagulated tubes, then stored under standardized conditions until analysis. Quantification of circulating inflammatory biomarkers, including hs-CRP, vWF, TGF-β, IL-1β, IL-2, IL-6, IL-8, and VEGF, was performed using Multiplex immunoassay technology (Luminex FlexMap 3D platform, Saluggia, Piemonte, Italy), in accordance with the manufacturer’s protocols. Subsequent days of inotropic support and days of hospitalization after CABG were evaluated. Acute kidney injury (defined by KDIGO criteria) and acute hepatic dysfunction (defined as ALT or AST > 3x upper limit of normal with either bilirubin > 2 mg/dL or coagulopathy with INR > 1.5 not attributable to anticoagulation) were also evaluated.

### 2.3. Statistical Analysis

Continuous variables are expressed as mean ± standard deviation or median and interquartile range, as appropriate. Categorial variables are expressed as absolute values and percentages. Non-parametric ANOVA with the Friedman test was used to compare atrial and ventricular arrhythmic burden between days 2, 3, and 4 post-CABG. Correlations between cardiovascular risk factors and post-procedural arrhythmic burden, as well as those between pre-procedural inflammatory biomarker levels and post-CABG PACs and PVCs burden, were assessed using the Spearman correlation test. All tests were two-sided, and a *p*-value < 0.05 was considered statistically significant. All data were computed using MedCalc 19.6.4 (MedCalc Software, Oostende, Belgium).

## 3. Results

**Figure 1:**
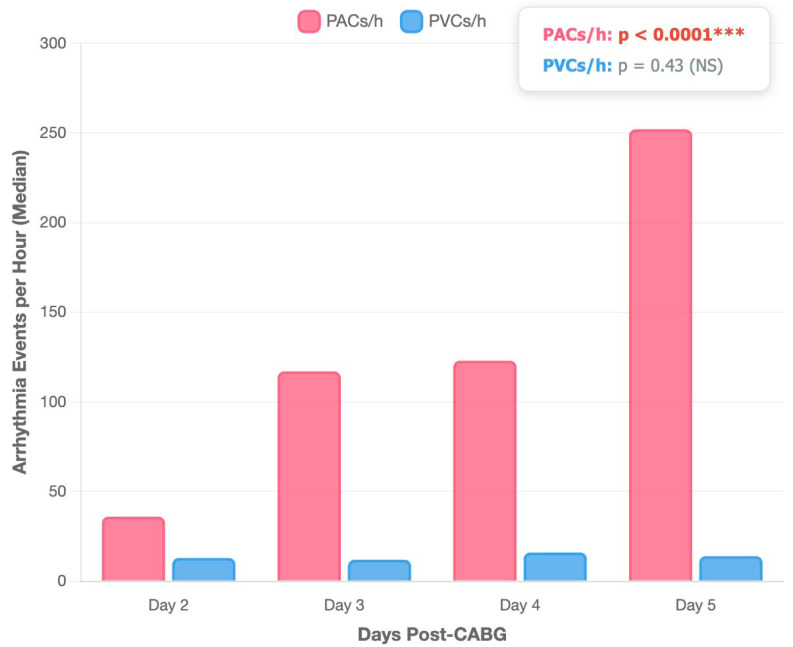
Kruskal–Wallis ANOVA test comparing temporal evolution of PACs and PVCs across post-operative days. PACs—premature atrial contractions; PVCs—premature ventricular contractions; CABG—coronary artery bypass grafting surgery; *** statistically significant; NS—not significant; *p*-values were obtained using the Friedman test.

A total of 102 patients treated by elective CABG for CCS were included in the analysis. The median age of the population was 60 (58–67) years, and 56.8% of participants were represented by the male gender. Overall, one-third of the population presented diabetes mellitus (33.3%), a body mass index (BMI) of 26.1 (24.3–30.8) kg/m^2^, and a median abdominal circumference of 102 (92.7–112.0) cm. Most of the patients were on chronic beta-blocker therapy (89.2%), RASS inhibitors (77.4%), and statins (90.1%). The median CHA_2_DS_2_-VASc score was 3 (2–4) points, and the population presented an intermediate SYNTAX II score of 23.5 (18.1–32.5). The median anteroposterior diameter of the left atrium was 37.5 mm [35.0–40.0 mm], while the median left atrial area was 19.0 cm^2^ [17.3–19.6 cm^2^]. Regarding left ventricular systolic function, the median LVEF 50% [45–55%]. Among the study population, 76.2% of patients were diagnosed with grade 1 diastolic dysfunction, while 23.8% presented with grade 2 diastolic dysfunction. A significant progressive increase in PACs was observed across the monitoring period (Kruskal–Wallis ANOVA, *p* < 0.0001). The median PACs frequency demonstrated a consistent upward trajectory from postoperative day 2 [36 events/hour (15–66)] to day 3 [117 events/hour (36–333)], remaining elevated on day 4 [123 events/hour (69–684)], and reaching peak values on day 5 [252 events/hour (141–645)]. In contrast, PVCs demonstrated temporal stability throughout the observation period (Kruskal–Wallis ANOVA, *p* = 0.43). Median PVC frequency remained relatively constant: day 2 [13 events/hour (8–32)], day 3 [12 events/hour (2–125)], day 4 [16 events/hour (0–102)], and day 5 [14 events/hour (2–38)], as Figure 1 shows.

### 3.1. Cardiovascular Risk Factors and Post-CABG Arrhythmic Burden

Postoperative PACs exhibited significant positive correlation with BMI (r = 0.25, 95% CI [0.05–0.43], *p* < 0.0001) and negative correlation with SYNTAX II score (r = −0.35, 95% CI [−0.51–0.16], *p* < 0.0001), as Table 1 demonstrates. The PAC’s ectopy was not correlated with prior beta-blocker (r = −0.01, 95% CI [−0.21–0.18], *p* = 0.8) or RAAS therapy (r = 0.10, 95% CI [−0.09–0.29], *p* = 0.2). Conversely, the post-CABG ventricular arrhythmic ectopy was positively correlated with the RAAS therapy (r = 0.28, 95% CI [0.08–0.45], *p* < 0.001), and negatively correlated with the LVEF (r = −0.39, 95% CI [−0.55–0.21], *p* < 0.0001) and with statin use (r = −0.28, 95% CI [−0.45–0.08], *p* < 0.001), as Table 1 details.

### 3.2. Pre-Procedural Inflammatory Biomarker Levels and Post-CABG PACs and PVCs Burden

Table 2 summarizes the pre-CABG plasma concentrations of the investigated biomarkers. Among them, vWF, TGF-β, and IL-8 levels demonstrated a positive correlation with PACs (r = 0.3, 95% CI [0.12–0.48], *p* < 0.001; r = 0.2, 95% CI [0.01–0.39], *p* = 0.03; r = 0.25, 95% CI [0.05–0.43], *p* < 0.001, respectively, Table 3). The strongest correlation between vWF, TGF-β, and PACs was observed on the third postoperative day following CABG, while the strongest correlation between IL-8 and PACs was documented on the second postoperative day. Conversely, preprocedural hs-CRP, TGF-β, IL-6, and IL-8 plasma levels (Table 3) presented a positive correlation with the PVCs (r = 0.67, 95% CI [0.55–0.77], *p* < 0.0001; r = 0.32, 95% CI [0.13–0.49], *p* < 0.0001; r = 0.21, 95% CI [0.01–0.03], *p* = 0.02; r = 0.69, 95% CI [0.57–0.78], *p* < 0.0001, respectively). The strongest correlation of hs-CRP and PVC was noticed on the second postoperative day, while IL-6 and IL-8 were noticed on the third postoperative day.

### 3.3. The Impact of Preoperative Inflammation on Post-CABG Outcomes

Patients who presented with acute kidney injury post-CABG (24.5%) were significantly older compared to patients without acute kidney injury (65 [58–73] years vs. 59 [57–66] years, *p* = 0.03). However, pre-procedural inflammatory biomarker levels (Table 4) were similar between patients with and without CABG-related acute kidney injury (all *p* > 0.05). In terms of acute liver failure (20.5%), pre-procedural biomarkers were similar between patients with and without CABG-related liver dysfunction (all *p* > 0.05, Table 4). Also, there was no relationship between preoperative inflammatory biomarkers and subsequent duration of inotropic support or days of hospitalization (all *p* > 0.05, Table 5).

## 4. Discussion

The main findings of the present study were that (1) preoperative plasma levels of vWF, TGF-β, and IL-8 are significantly correlated with increased postoperative atrial ectopy, and (2) preoperative concentrations of hs-CRP, TGF-β, IL-6, and IL-8 are associated with a higher ventricular ectopy, reinforcing the potential for precision-targeted antiarrhythmic therapies, with a personalized approach focusing on specific cytokines. However, (3) none of the tested biomarkers in the present study could predict other postoperative outcomes, such as acute kidney injury, acute liver failure, duration of inotropic support, and days of hospitalization.

### 4.1. Cardiovascular Risk Factors and Post-CABG PACs and PVCs Burden

Despite recent improvements in the management of CCS, the burden of cardiac arrhythmias following CABG remains substantial, often leading to adverse cardiovascular outcomes. As a result, identifying individual postoperative arrhythmic profiles could help distinguish patients at higher risk for developing these arrhythmias, enabling more targeted monitoring strategies and closer follow-up of this population.

According to the present study findings, patients with lower SYNTAX II scores tend to present a higher burden of PACs after CABG. Differing revascularization approaches with a less aggressive surgical approach, together with partial revascularization, might leave areas of borderline ischemia that could trigger ectopic beats [18]. These findings suggest that patients with reduced complexity of coronary anatomy should receive more vigilant monitoring for atrial ectopy. Moreover, it changes the paradigm of current risk stratification, which can inadequately capture arrhythmic risk as it tends to focus on patients with complex diseases. Most of the studies have evaluated the effect of various cardiovascular risk factors on the incidence of AF after CABG, which is the expression of a more advanced stage of atrial cardiomyopathy [19,20]. However, PACs have recently been associated with an increased risk of incident and stroke, and there is growing evidence of their potential role as a signaling marker of atrial cardiomyopathy [4].

Chronic use of beta-blockers or RAAS inhibitors does not significantly affect the burden of postoperative PACs in our population. One study on the previous beta-blockers’ effect on atrial arrhythmic burden showed no significant association with postoperative AF [21]. Since the protective effects of beta-blockers in reducing the atrial arrhythmic burden were demonstrated in patients with a history of myocardial infarction or congestive heart failure, the present findings suggest the complex role of modulating the autonomic nervous system in different clinical backgrounds [22]. The heterogeneous evidence in the literature highlights the complex interplay of physiological mechanisms that might explain these findings. Surgical stress can create a unique physiological environment that may neutralize the expected effects of beta-blockers and RASS inhibitors, while acute autonomic and hormonal changes during cardiac surgery can override chronic medication effects [23].

### 4.2. Pre-Procedural Inflammatory Profile and Post-CABG PACs and PVCs

While the majority of studies have focused on post-operative inflammatory responses and cardiac arrhythmias following CABG, this study introduces novel evidence that preoperative plasma levels of vWF, TGFβ, and IL-8 are significantly correlated with increased postoperative atrial ectopy, and that preoperative concentrations of hs-CRP, TGF-β, IL-6, and IL-8 are associated with a higher burden of ventricular ectopy. These findings support the hypothesis that a heightened basal inflammatory status may elevate the risk of arrhythmic burden after CABG.

Despite the potential clinical significance, the current literature presents a notable gap in understanding the direct correlation between preoperative inflammatory biomarkers and postoperative arrhythmic burden. Preoperative plasma levels of IL-6, hs-CRP, and vWF exhibit strong and consistent correlations with postoperative atrial burden in CABG patients, while limited evidence exists for ventricular ectopy prediction, and fewer studies investigate biomarkers like TGF-β or IL-8 [24,25].

The potential role of endothelial damage, reflected by alterations in VWF levels, in the pathogenesis of cardiac arrhythmias was investigated on intracardiac specimens, specifically from the left atrial appendage (LAA), in patients undergoing CABG [26]. Kaireviciute and collaborators demonstrated that elevated VWF expression in LAA tissue serves as a significant predictor of postoperative AF, underscoring the importance of tissue-specific expression of pathophysiologic markers in the development of postoperative arrhythmias [26]. On the other hand, another study demonstrated that the concentrations of vWF were unrelated to postoperative AF, while intracardiac levels of IL-6 were associated with AF [27], emphasizing the heterogeneity and complexity of the inflammatory system in the development of cardiac arrhythmias. This variability highlights the multifaceted nature of inflammation and its intricate relationship with arrhythmogenesis, emphasizing the need for future research.

TGF-β has been shown to contribute to the development and progression of atrial arrhythmias, primarily via established pro-fibrotic mechanisms, particularly through structural remodeling [28]. However, its role in the pathogenesis of postoperative arrhythmic burden in patients undergoing CABG has not been extensively investigated, with the majority of existing data focusing on the examination of TGF-β concentrations within cardiac tissue [29]. Our findings revealed a critical link between preoperative TGF-β plasma levels and subsequent atrial and ventricular ectopy, highlighting the complex interplay between the inflammatory process and arrhythmogenesis. TGF-β plays a pivotal role in inflammatory signaling and tissue remodeling, which creates predispositions to cardiac electrical disturbances [30]. Potential mechanisms for preoperative TGF-β-related arrhythmogenesis include inflammation-induced ion channel alterations, cardiac tissue remodeling, and modified gap junction communication [30]. The positive association with atrial and ventricular ectopy suggests a systemic inflammatory influence on cardiac electrical properties. Therefore, TGF-β levels offer a promising tool for identifying patients at higher risk for postoperative arrhythmias, implementing targeted preventive strategies, and guiding individualized perioperative management.

**Figure 2:**
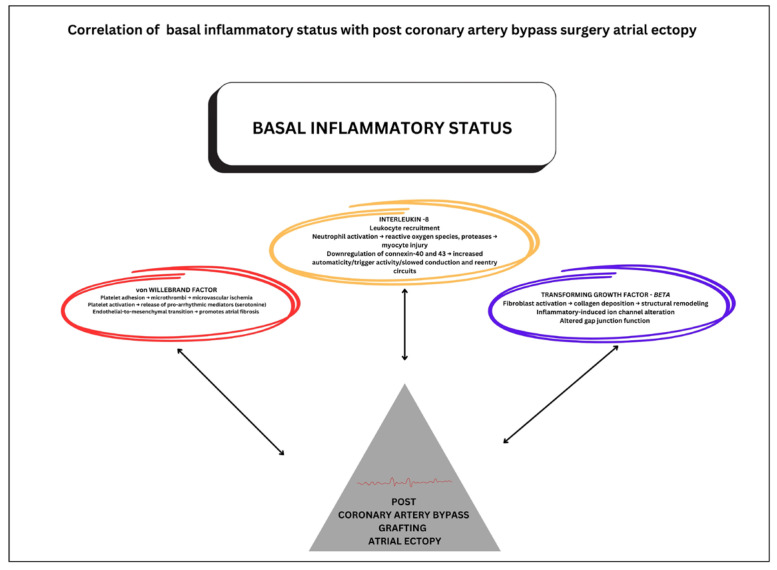
Mechanisms linking preoperative inflammatory status to post-coronary artery bypass grafting atrial ectopy.

Additionally, elevated vWF promotes platelet adhesion, leading to microthrombi formation and consequent microvascular ischemia, contributing to atrial arrhythmogenesis [31]. Moreover, vWF facilitates endothelial-to-mesenchymal transition, accelerating atrial structural remodeling through fibrotic transformation [32]. Elevated preoperative plasma concentrations of IL-8 represent a potential mechanistic contributor to post-CABG atrial ectopic activity, since IL-8 mediates neutrophil chemotaxis and activation, resulting in oxidative stress and protease release within atrial tissue [33]. These alterations establish an electrophysiological substrate characterized by enhanced automaticity, triggered activity, and impaired intercellular electrical coupling. Finally, these findings not only enhance our understanding of the inflammatory pathways involved in arrhythmogenesis but also underscore the need for further investigation into TGF-β, vWF, and IL-8 roles as therapeutic targets or prognostic indicators for this population. Figure 2 depicts the interplay between inflammatory mediators, platelet activation, and altered atrial electrophysiology that potentially underlies post-CABG atrial ectopy.

A schematic representation of the potential pathophysiological mechanisms connecting pre-operative inflammatory status to the development of atrial ectopy following coronary artery bypass grafting surgery illustrates three key mechanisms: (red) von Willebrand Factor pathway-involving platelet adhesion, microthrombosis, microvascular ischemia, and endothelial-to-mesenchymal transition promoting atrial fibrosis; (orange) Interleukin-8 pathway-characterized by neutrophil activation, reactive oxygen species production, proteases, myocyte injury, and downregulation of connexin-40 and −43 leading to altered conduction and reentry circuits; and (blue) transforming growth factor-β pathway-mediating fibroblast activation, collagen deposition, structural remodeling, and inflammatory-induced ion channel alterations affecting gap junction function.

**Figure 3:**
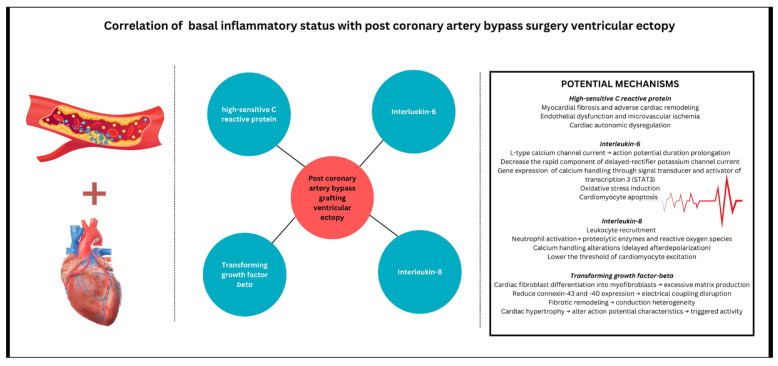
Inflammatory biomarkers and their mechanistic pathways in post-coronary artery bypass grafting surgery ventricular ectopy.

Biomarker relationships with postoperative ventricular ectopy remain underexplored since studies have predominantly focused on atrial arrhythmias. In the present cohort of CABG patients, preoperative hs-CRP, IL-6, and IL-8 levels demonstrated a significant association with ventricular ectopic activity. This is consistent with findings showing that IL-6 and IL-8 play a critical role in the prolongation of action potential duration (APD) and related ventricular arrhythmogenesis [34]. Ogawa et al. were the first to demonstrate that IL-6 can increase L-type calcium channel currents, leading to APD prolongation [34]. Additionally, IL-6 has been shown to decrease the rapid component of the delayed-rectifier potassium channel current, contributing to the complex alteration in cardiac electrophysiology [35]. Furthermore, direct administration of IL-6 to guinea pigs acutely prolongs the QT interval, which was blocked by the IL-6 receptor blocker [36]. These observations are reinforced by recent clinical data, which identified circulating IL-6 as a risk factor for QT interval prolongation in a large patient cohort [37]. This strengthens the hypothesis that IL-6 may have a significant arrhythmogenic role in the general population, with baseline inflammatory status potentially exerting an even greater influence on the postoperative burden of ventricular arrhythmias. Additionally, hs-CRP-induced endothelial dysfunction and microvascular ischemia can further exacerbate ventricular ectopy by promoting localized areas of hypoperfusion and ischemia-induced heterogeneity in repolarization [38]. TGF-β, a key mediator of fibrosis, contributes to excessive extracellular matrix production and has also been shown to reduce the expression of connexins-43 and -40, critical components of gap junctions in the myocardium [39]. This downregulation may lead to impaired electrical coupling and conduction heterogeneity, thereby facilitating the development of ventricular ectopy (Figure 3)

The diagram illustrates the relationship between pre-operative inflammatory biomarkers and the development of ventricular ectopy following coronary artery bypass grafting. Four key inflammatory mediators are depicted: highly sensitive C-reactive protein, interleukin-6, transforming growth factor-β, and interleukin-8. The right panel details the potential mechanisms through which these biomarkers contribute to ventricular arrhythmogenesis, including: (1) highly sensitivity C-reactive protein pathway-promoting myocardial fibrosis, adverse cardiac remodeling, endothelial dysfunction, microvascular ischemia, and cardiac autonomic dysregulation; (2) Interleukin-6 pathway-affecting L-type calcium channel currents, action potential duration prolongation, delayed-rectifier potassium channel modulation, calcium transient alterations, oxidative stress induction, and cardiomyocyte apoptosis; (3) Interleukin-8 pathway-inducing neutrophil activation, proteolytic enzyme release, calcium handling alterations, and lowering the threshold for cardiomyocyte excitation; and (4) transforming growth factor-β pathway-driving cardiac fibroblast differentiation, excessive matrix production, electrical coupling disruption, conduction heterogeneity, and cardiac hypertrophy.

The progression from understanding cytokines as inflammatory markers to exploring their potential therapeutic applications represents a significant evolution in cardiovascular research, and recent evidence promises more nuanced, personalized inflammatory management strategies. The safety and tolerability of low-dose cytokine therapy were demonstrated in a randomized, double-blind, placebo-controlled trial involving patients with stable ischemic heart disease [40]. These promising results suggest a potentially significant advancement in the personalized treatment and prevention of cardiac arrhythmias in this patient population.

### 4.3. Atrial Cardiomyopathy and the Relationship with Premature Atrial Contractions in the Context of Coronary Artery Bypass Grafting Surgery

The concept of atrial cardiomyopathy, defined as complex structural, architectural, contractile, or electrophysiological changes affecting the atria with the potential to produce clinically relevant manifestations, represents a new paradigm in understanding the pathogenesis of thromboembolic events and atrial fibrillation, as previously mentioned [4,41].

In the context of the present study results, which demonstrate significant correlations between preoperative inflammatory biomarkers (vWF, TGF-β, IL-8) and postoperative PAC burden after CABG, a complex pathophysiological mechanism emerges linking systemic inflammation to atrial remodeling. The identification in this study of the association between preoperative vWF and TGF-β with postoperative PACs suggests that these biomarkers may reflect a vulnerable atrial substrate predisposed to developing atrial cardiomyopathy. TGF-β1, a profibrotic cytokine, increases atrial fibrosis and vulnerability to atrial arrhythmias [28]. This relationship underscores the importance of preoperative assessment of inflammatory status as a predictive tool for identifying patients at increased risk of developing significant post-CABG atrial ectopy. Furthermore, these findings open new research horizons in understanding the intricate relationship between preoperative inflammatory milieu and postoperative atrial cardiomyopathy development.

### 4.4. Clinical Implications of Preoperative Signature Inflammation

Inflammatory activation is progressively recognized as a significant risk factor for cardiac arrhythmias, revealing the complex pathophysiological mechanism linking systemic inflammation to electrophysiological vulnerability. However, baseline proinflammatory status is still neglected, and circulating cytokines are not routinely assessed in clinical practice. This study provides new insights into the role of basal inflammatory profile in cardiac arrhythmogenesis, highlighting the impact of preoperative inflammatory status on atrial and ventricular ectopy in a specific subgroup of patients with CCS and CABG. Clinically, this suggests the potential for a targeted screening approach to identify high-risk patients before surgery. Practical implications include developing personalized perioperative monitoring protocols, considering early preventive interventions, and tailoring patient management based on individual inflammatory profiles. By integrating these inflammatory signatures into preoperative risk assessment, clinicians can potentially reduce arrhythmic complications and optimize patient outcomes through more precise, individualized cardiac surgical care.

### 4.5. Limitations and Future Directions

The results’ applicability to larger populations may be limited by the single-center design. Also, statistical analysis employed Spearman’s rank correlation to assess associations between preoperative inflammatory markers and postoperative arrhythmic events. While multivariate analysis would be valuable for adjusting for potential confounders, the event-to-variable ratio in our cohort would limit the statistical reliability of such models. Our findings provide hypothesis-generating data that warrant validation in larger cohorts with adequate statistical power for multivariable modeling. The use of serial 24-h Holter monitoring for four consecutive post-operative days enabled a comprehensive arrhythmia surveillance strategy that captures the critical early post-operative period when inflammatory responses peak and arrhythmic risk is highest. While our study focused on PACs and PVCs as discrete arrhythmic events, we recognize that postoperative AF represents another important inflammatory-mediated complication. The current analysis provides foundational data on the inflammation-arrhythmia relationship that will inform future investigations examining more complex arrhythmic patterns.

## 5. Conclusions

The present study demonstrates that specific preoperative inflammatory biomarkers are strongly related to an increased risk of postoperative atrial and ventricular ectopy, pointing towards a potential role in identifying patients at risk for developing subsequent arrhythmias among patients with CABG. Moreover, these results not only advance our understanding of inflammatory mechanisms in cardiac surgery but also provide a foundation for future research aimed at developing targeted interventions to reduce the burden of arrhythmic complications.

## Figures and Tables

**Table 1:** Correlations between cardiovascular risk factors and post-coronary artery bypass surgery (CABG) atrial and ventricular ectopic beats.

Post-CABG Arrhythmic Burden	Parameters	Spearman r	95% CI	*p*-Value
PACs	Age	0.12	−0.07 and 0.31	0.20
	Body mass index	0.25	0.05 and 0.43	**<0.001**
	Abdominal circumference	0.24	0.04 and 0.42	**0.01**
	Beta-blocker	−0.01	−0.21 and 0.18	0.80
	Renin-angiotensin-aldosterone inhibitors	0.10	−0.09 and 0.29	0.29
	Statins	−0.02	−0.22 and 0.17	0.80
	CHA_2_DS_2_-VASc score	0.04	−0.15 and 0.24	0.63
	SYNTAX II score	−0.35	−0.51 and 0.16	**<0.0001**
	Left ventricle ejection fraction	0.10	−0.05 and 0.33	0.13
	Left atrium area	0.06	−0.30 and 0.41	0.74
PVCs	Age	−0.002	−0.20 and 0.19	0.90
	Body mass index	−0.04	−0.23 and 0.16	0.68
	Abdominal circumference	−0.14	−0.33 and 0.06	0.15
	Beta-blocker	−0.13	−0.32 and 0.07	0.1
	Renin-angiotensin-aldosterone inhibitors	0.28	0.08 and 0.45	**<0.001**
	Statins	−0.28	−0.45 and 0.08	**<0.001**
	CHA_2_DS_2_-VASc score	0.01	−0.18 and 0.21	0.84
	SYNTAX II score	0.07	−0.12 and 0.27	0.44
	Left ventricle ejection fraction	−0.39	−0.55 and 0.21	**<0.0001**

*p*-values were obtained using the Spearman test. Bold values indicate the parameter for which *p*-values were statistically significant (*p* < 0.05). CI—confidence interval; PACs—premature atrial contractions; PVCs—premature ventricular contractions.

**Table 2:** Pre-coronary artery bypass surgery (CABG) plasma concentrations of the inflammatory biomarkers.

Inflammatory Biomarkers	Median Pre-CABG Plasma Level (n = 102)
Highly sensitive C-reactive protein (mg/L)	1.67 (0.52–5.52)
Von Willebrand factor (IU/dL)	1.78 (1.45–3.14)
Transforming growth factor-β (ng/mL)	68,680 (52,684–74,835)
Interleukin-1b (pg/mL)	12.86 (5.45–29.66)
Interleukin-2 (pg/mL)	5.73 (2.97–14.09)
Interleukin-6 (pg/mL)	8.38 (2.80–18.48)
Interleukin-8 (pg/mL)	7.61 (3.31–21.29)
Vascular endothelial growth factor (pg/mL)	631.60 (422.3–1025)

**Table 3:** Correlation of pre-procedural inflammatory biomarker levels and post-coronary artery bypass surgery (CABG), premature atrial contractions (PACs), and premature ventricular contractions (PVCs).

Post-CABG Arrhythmic Burden	Parameters	Spearman r	95% CI	*p*-Value
PACs	Highly sensitive C-reactive protein	0.14	−0.06–0.33	0.10
	Von Willebrand factor	0.3	0.12–0.48	**<0.001**
	Transforming growth factor β	0.2	0.01–0.39	**0.03**
	Interleukin-1b	−0.1	−0.29–0.09	0.20
	Interleukin-2	−0.09	−0.28–0.11	0.30
	Interleukin-6	−0.14	−0.33–0.06	0.10
	Interleukin-8	0.25	0.05–0.43	**<0.001**
	Vascular endothelial growth factor	−0.11	−0.30–0.08	0.20
PVCs	High-sensitive C-reactive protein	0.67	0.55–0.77	**<0.0001**
	Von Willebrand factor	0.08	−0.11–0.28	0.30
	Transforming growth factor β	0.32	0.13–0.49	**<0.0001**
	Interleukin-1b	0.16	−0.03–0.35	0.09
	Interleukin-2	0.07	−0.12–0.27	0.40
	Interleukin-6	0.21	0.01–0.3	**0.02**
	Interleukin-8	0.69	0.57–0.78	**<0.0001**
	Vascular endothelial growth factor	0.13	−0.06–0.32	0.10

*p*-values were obtained using the Spearman test. Bold values indicate the parameter for which *p*-values were statistically significant (*p* < 0.05). CI—confidence interval; PACs—premature atrial contractions; PVCs—premature ventricular contractions.

**Table 4:** The impact of postoperative arrhythmic burden on post-CABG-related acute renal and hepatic failure.

Parameters	Total (n = 102)	Acute Kidney Failure (n = 25)	Without Acute Kidney Failure (n = 77)	*p*-Value
Age (years)	60 (58–67)	65 (58–73)	59 (57–66)	**0.03**
Abdominal circumference (cm)	102 (92.7–112)	104 (95.2–113)	100 (92–113)	0.50
Male gender (n, %)	58 (56%)	17 (68%)	59 (76%)	0.50
Highly sensitive C-reactive protein (mg/L)	1.67 (0.52–5.52)	3.26 (0.52–7.44)	1.42 (0.52–5.22)	0.25
Von Willebrand factor (IU/dL)	1.78 (1.45–3.14)	1.59 (1.09–2.79)	1.85 (1.57–3.23)	0.08
Transforming growth factor-β (ng/mL)	68,680 (52,684–74,835)	68,680 (51,657–72,165)	70,670 (52,684–85,758)	0.35
Interleukin-1b (pg/mL)	12.86 (5.45–29.66)	13.04 (5.04–28.11)	12.79 (6.49–31.00)	0.61
Interleukin-2 (pg/mL)	5.73 (2.97–14.09)	6.35 (2.17–14.34)	5.12 (3.12–13.19)	0.86
Interleukin-6 (pg/mL)	8.38 (2.80–18.48)	10.58 (3.78–17.71)	7.08 (2.80–18.48)	0.71
Interleukin-8 (pg/mL)	7.61 (3.31–21.29)	4.37 (3.10–17.17)	7.88 (3.31–23.76)	0.27
Vascular endothelial growth factor (pg/mL)	631.60 (422.3–1025)	674 (380.7–1052)	621 (422.30–1038)	0.95
Parameters	Total (n = 102)	Acute hepatic failure (n = 21)	Without acute hepatic failure (n = 81)	*p*-value
Age (years)	60 (58–67)	64 (58–70)	59 (57–67)	0.10
Abdominal circumference (cm)	102 (92.7–112)	99 (92.5–115)	102 (92.5–111)	0.70
Male gender (n, %)	58 (56%)	19 (90.4%)	57 (70.3%)	0.10
Highly sensitive C-reactive protein (mg/L)	1.67 (0.52–5.52)	1.67 (0.49–7.44)	1.67 (0.53–5.22)	0.71
Von Willebrand factor (IU/dL)	1.78 (1.45–3.14)	1.75 (1.45–2.27)	1.85 (1.45–3.44)	0.51
Transforming growth factor-β (ng/mL)	68,680 (52,684–74,835)	63,019 (52,155–85,758)	70,670 (52,684–73,697)	0.90
Interleukin-1b (pg/mL)	12.86 (5.45–29.66)	13.04 (10.50–33.86)	12.79 (5.45–29.21)	0.17
Interleukin-2 (pg/mL)	5.73 (2.97–14.09)	7.01 (3.79–17.35)	4.57 (2.52–13.19)	0.10
Interleukin-6 (pg/mL)	8.38 (2.80–18.48)	10.81 (4.40–22.18)	6.76 (2.80–18.07)	0.12
Interleukin-8 (pg/mL)	7.61 (3.31–21.29)	4.44 (3.10–15.23)	7.88 (3.37–23.76)	0.30
Vascular endothelial growth factor (pg/mL)	631.60 (422.3–1025)	713.80 (526.6–1092.0)	621 (401.5–949.2)	0.25

Quantitative data are expressed as median (interquartile range). Categorical data are expressed as numbers (percentages). *p*-values were obtained using the Mann–Whitney U test for continuous variables and Fisher’s exact test for categorical data. Bold values indicate the parameters for which differences between groups were statistically significant (*p* < 0.05).

**Table 5:** The impact of postoperative arrhythmic burden on subsequent days of inotropic support and days of hospitalization after CABG.

Post-CABG Outcome	Parameters	Spearman r	95% CI	*p*-Value
Days of inotropic support	Highly sensitive C-reactive protein	0.008	−0.19–0.20	0.90
	Von Willebrand factor	−0.10	−0.29–0.09	0.29
	Transforming growth factor-β	0.001	−0.19–0.20	0.90
	Interleukin-1b	−0.02	−0.22–0.17	0.83
	Interleukin-2	−0.002	−0.20–0.19	0.90
	Interleukin-6	0.06	−0.13–0.26	0.49
	Interleukin-8	0.06	−0.13–0.26	0.48
	Vascular endothelial growth factor	0.005	−0.19–0.20	0.90
Days of hospitalization	High-sensitive C reactive protein	−0.01	−0.21–0.18	0.87
	Von Willebrand factor	−0.04	−0.24–0.15	0.63
	Transforming growth factor-β	−0.14	−0.33–0.05	0.14
	Interleukin-1b	0.06	−0.14–0.25	0.55
	Interleukin-2	0.05	−0.14–0.25	0.50
	Interleukin-6	0.04	−0.15–0.24	0.63
	Interleukin-8	0.07	−0.12–0.27	0.43
	Vascular endothelial growth factor	0.09	−0.11–0.28	0.36

*p*-values were obtained using the Spearman test. CI—confidence interval. PACs—premature atrial contractions.

## Data Availability

The datasets used and analyzed during the current study are available from the corresponding author upon request.

## Abbreviations

AF—atrial fibrillation; APD—action potential duration; CABG—coronary artery bypass grafting; CCS—chronic coronary syndrome; hs-CRP—highly sensitive C-reactive protein; IL—interleukin; LEVF—left ventricular ejection fraction; PACs—premature atrial contractions; PVCs—premature ventricular complexes; RAAS—renin-angiotensin-aldosterone system; TGF-β—transforming growth factor-beta; VEGF—vascular endothelial growth factor; vWF—von Willebrand factor.
